# Semantic Interoperability of Electronic Health Records: Systematic Review of Alternative Approaches for Enhancing Patient Information Availability

**DOI:** 10.2196/53535

**Published:** 2024-04-25

**Authors:** Sari Palojoki, Lasse Lehtonen, Riikka Vuokko

**Affiliations:** 1 Department of Steering of Healthcare and Social Welfare, Ministry of Social Affairs and Health, Helsinki, Finland; 2 Diagnostic Center, Helsinki University Hospital District, Helsinki, Finland

**Keywords:** electronic health record, health records, EHR, EHRs, semantic, health care data, semantic interoperability, interoperability, standardize, standardized, standardization, cross-border data exchange, systematic review, synthesis, syntheses, review methods, review methodology, search, searches, searching, systematic, data exchange, information sharing, ontology, ontologies, terminology, terminologies, standard, standards, classification, PRISMA, data sharing, Preferred Reporting Items for Systematic Reviews and Meta-Analyses

## Abstract

**Background:**

Semantic interoperability facilitates the exchange of and access to health data that are being documented in electronic health records (EHRs) with various semantic features. The main goals of semantic interoperability development entail patient data availability and use in diverse EHRs without a loss of meaning. Internationally, current initiatives aim to enhance semantic development of EHR data and, consequently, the availability of patient data. Interoperability between health information systems is among the core goals of the European Health Data Space regulation proposal and the World Health Organization’s *Global Strategy on Digital Health 2020-2025*.

**Objective:**

To achieve integrated health data ecosystems, stakeholders need to overcome challenges of implementing semantic interoperability elements. To research the available scientific evidence on semantic interoperability development, we defined the following research questions: What are the key elements of and approaches for building semantic interoperability integrated in EHRs? What kinds of goals are driving the development? and What kinds of clinical benefits are perceived following this development?

**Methods:**

Our research questions focused on key aspects and approaches for semantic interoperability and on possible clinical and semantic benefits of these choices in the context of EHRs. Therefore, we performed a systematic literature review in PubMed by defining our study framework based on previous research.

**Results:**

Our analysis consisted of 14 studies where data models, ontologies, terminologies, classifications, and standards were applied for building interoperability. All articles reported clinical benefits of the selected approach to enhancing semantic interoperability. We identified 3 main categories: increasing the availability of data for clinicians (n=6, 43%), increasing the quality of care (n=4, 29%), and enhancing clinical data use and reuse for varied purposes (n=4, 29%). Regarding semantic development goals, data harmonization and developing semantic interoperability between different EHRs was the largest category (n=8, 57%). Enhancing health data quality through standardization (n=5, 36%) and developing EHR-integrated tools based on interoperable data (n=1, 7%) were the other identified categories. The results were closely coupled with the need to build usable and computable data out of heterogeneous medical information that is accessible through various EHRs and databases (eg, registers).

**Conclusions:**

When heading toward semantic harmonization of clinical data, more experiences and analyses are needed to assess how applicable the chosen solutions are for semantic interoperability of health care data. Instead of promoting a single approach, semantic interoperability should be assessed through several levels of semantic requirements A dual model or multimodel approach is possibly usable to address different semantic interoperability issues during development. The objectives of semantic interoperability are to be achieved in diffuse and disconnected clinical care environments. Therefore, approaches for enhancing clinical data availability should be well prepared, thought out, and justified to meet economically sustainable and long-term outcomes.

## Introduction

Over the past 2 decades, there has been growing interest in digital technologies and eHealth integration into national health care systems to promote health [1]. The World Health Organization (WHO) has launched the *Global Strategy on Digital Health 2020-2025* [2]. To implement digital health strategy objectives, a toolkit was set up to help countries to integrate eHealth into their health care systems [3]. The objectives of the WHO strategy include standards for interoperability. Another current large-scale international initiative is the European Health Data Space (EHDS) regulation proposal. EHDS is a health-specific ecosystem comprised of rules, common standards and practices, infrastructures, and a governance framework. It supports the use of health data for better health care delivery, research, innovation, and policy making. Moreover, it aims at empowering patients through increased digital access to and control of their personal health data [3-6].

Interoperability ensures health data availability and use. It is the ability of different organizations and professionals to interact and share information according to standards of data transfer and common protocols that support data exchange [4-8]. In clinical context, interoperable electronic health records (EHRs) help health care practitioners gather, store, and communicate essential health information reliably and securely across care settings. This aims to guarantee coordinated and patient-centered care while creating many efficiencies in the delivery of health care [9]. EHRs use health-related information pertinent to an individual patient, whereas registries are mainly focused on population management and are designed to obtain information on predefined health outcomes data and data for public health surveillance, for example. Although technological possibilities for using various types of data grow, new demands are placed on data quality and usability and, consequently, on interoperability [5,10,11].

Moreover, semantic interoperability enhances the unambiguous representation of clinical concepts, supported by the use of international standard reference systems and ontologies. Since there are different types of health information, such as data from EHRs, patient registries, genomics data, and data from health applications, the development of international data standardization, common guidelines, and recommendations are needed [4-8]. Without applying appropriate semantic standards, such as domain-relevant terminologies, interoperability will be limited. This may diminish the availability and potential value of data. The various parties involved have to address the importance of shared digital health standards and especially semantic interoperability features [12-15]. In the clinical context, interoperability is required to enhance the quality, efficiency, and effectiveness of the health care system by providing information in the appropriate format whenever and wherever it is needed by eliminating unnecessary replication [16].

Therefore, our study aims to provide readers with up-to-date information about the different types of approaches to resolve semantic interoperability in EHRs specifically and to summarize the benefits of these choices. We aimed to research the topic with an emphasis on patient data availability and use. Our research questions were as follows: What are the key elements of and approaches for building semantic interoperability integrated in EHRs? What kinds of goals are driving the development? and What kinds of clinical benefits are perceived following this development?

## Methods

### Methodological Framework

With our research questions as a starting point, we set out to perform a systematic literature review of semantic interoperability. Regarding different layers of interoperability, legal interoperability ensures overcoming potential barriers for data exchange. Interoperability agreements are made binding via international- or national-level legislation and via bilateral and multilateral agreements. Organizational interoperability defines, for example, business goals and processes. Semantic interoperability ensures that the precise meaning of exchanged information is understandable by any other application. It enables systems to combine received information with other information resources and process it in a meaningful manner. Technical interoperability covers various issues of linking computer systems and services, such as open interfaces, data integration, data presentation and exchange, accessibility, and security services [6,7].

**Figure 1.**
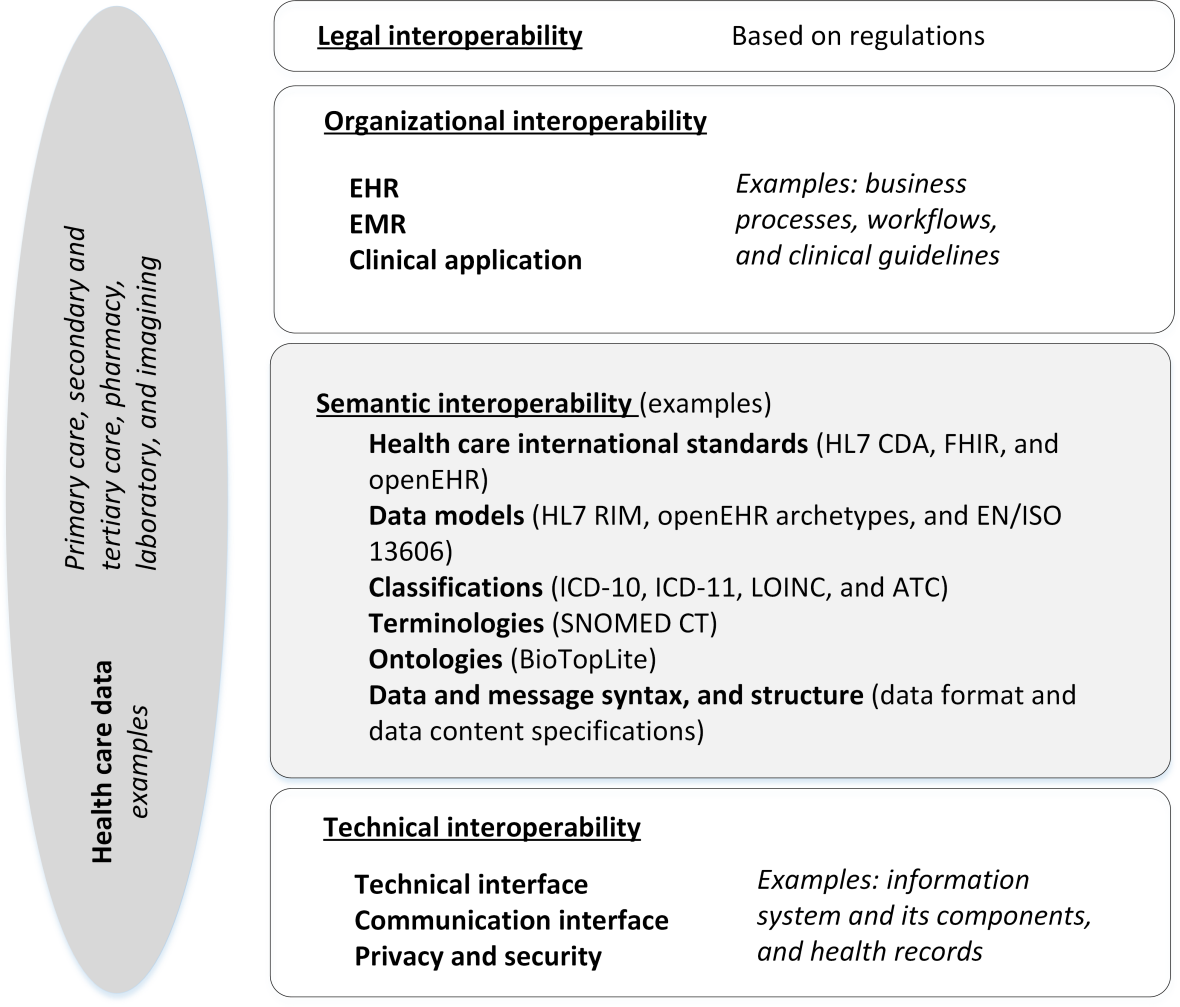
Our framework for defining semantic interoperability elements for conducting the literature search and guiding our study design. ATC: Anatomical Therapeutic Chemical; CDA: Clinical Document Architecture; EHR: electronic health record; EMR: electronic medical record; FHIR: Fast Health Interoperability Resources; HL7: Health Level 7; *ICD-10: International Classification of Diseases, Tenth Revision*; *ICD-11: International Classification of Diseases, 11th Revision*; LOINC: Logical Observation Identifiers Names and Codes; RIM: reference information model; SNOMED CT: Systematized Nomenclature of Medicine Clinical Terminology.

For the study design, we first defined our core concepts to refine the literature search strategy. The scope of the review was semantic interoperability, that is, organizational, legal, and technical interoperability were excluded [7]. Semantic interoperability was apprehended based on the European Interoperability Framework (EIF) that provides a common set of principles and guidance for the design and development of interoperable digital services. In the EIF, semantic interoperability covers both semantic and syntactic aspects. The semantic aspect refers to the meaning of data elements and their relationships, whereas the syntactic aspect refers to the format of the information to be exchanged. With semantic interoperability, it is ensured that data can be shared in such a way that the meaning of data does not change [7,15,17,18]. There are also other models for analyzing interoperability layers [18]. For example, in comparison to the European approach [7], the Healthcare Information and Management Systems Society defines 4 levels of interoperability for health care technology: foundational, structural, semantic, and organizational [19,20]. Since the EIF is a well-established and largely applied framework [6], we chose the EIF definitions to primarily guide our review framework, as illustrated in Figure 1. Our review deals with semantic interoperability, which is highlighted in gray in the figure. Thus, we did not analyze, for example, standards that are related to processes or information quality.

As shown in Figure 1, processing, storing, and exchanging health care data in EHRs and between EHRs or other clinical applications is, for example, governed and regulated at the legal layer. To continue, processes and workflows regarding information exchange are arranged at the organizational interoperability layer and resolved in the technical layer, for example, according to the principles of data protection and information security. To illustrate the point, for example, the EHDS proposal suggests that compliance with essential requirements on interoperability and data security may be demonstrated by the manufacturers of EHR systems through the implementation of common specifications. To that end, implementation can be grounded on common specifications, such as data sets, coding systems, technical specifications, standards, and profiles for data exchange, as well as requirements and principles related to security, confidentiality, integrity, patient safety, and he protection of personal data and so on [6].

The semantic interoperability layer in Figure 1 covers various approaches to resolve interoperability issues, such as more established international or domain-specific health care classifications, clinical terminologies, and ontologies and applications of international standards for EHRs. In Figure 1, we provided some examples to illustrate various semantic aspects, but this is not an exhaustive list. Similarly, for other interoperability levels, real-world examples were given. Based on the EIF, semantic interoperability also covers syntactic features, such as data format and, for example, structured data content. We identified these key features of semantic interoperability based on previous research [8,16-19,21]. In our framework, a data model is a generic concept that describes various applications of data models from a reference information model (RIM) to a clinical information model. Data models define structures and semantics for storing, exchanging, querying, and processing health care data. Clinical information models can be implemented in an EHR, for example, as archetypes and templates, whereas RIMs refer to standards-based approaches to enable health care documentation and messages, such as the Health Level 7 (HL7) RIM or the International Organization for Standards’ EN/ISO 13606 standard for EHR communication [19,22]. When designing EHRs, for semantic interoperability, a dual-level method can be applied to represent both information and knowledge levels of interoperability requirements, properties, and structures for data. This approach is used, for example, for representing the dual levels of knowledge by an archetype model and information structures by the chosen RIM [16,21,22].

### Study Design

In the design of the review, we applied the Cochrane review protocol [23] to ensure the scientific reliability and validity of our review (Checklist 1). The search strategy (see Textbox 1) was defined based on the framework for semantic interoperability presented in Figure 1. We performed the search in the PubMed database in December 2022. To conduct a systematic literature review, PubMed is regarded as a comprehensive database [24]. Therefore, no further data searches were performed. We documented the search so that it can be reproduced (see Textbox 1). The search resulted in 131 unique articles. One article was removed because it did not include an abstract, and 1 was removed because it was not in English. In total, the authors screened 129 articles.

Textbox 1. **Search strategy and filters used.**

- Search terms: (((((EHR) OR (EMR)) OR (“Electronic Health Record”)) OR (“Electronic Medical Record”)) AND (((((“Semantic interoperability”) OR ((“data model”) AND (“Semantic interoperability”))) OR ((((“classification”) OR (ontology)) OR (terminology)) AND (“Semantic interoperability”))) OR (((“data content”) OR (“data format”)) AND (“Semantic interoperability”))) OR ((“Semantic interoperability”) AND (standard)))
- Filters used: abstract, full text, and English

The research team first screened all the remaining articles by title and abstract from January to March 2023. After the first test reading, the researchers discussed the inclusion and exclusion criteria and coherence of the understanding. Researchers were blinded and performed the analysis independently based on the inclusion and exclusion criteria and then compared the results. Selecting the same alternative created a match. Choosing a different alternative or failing to recognize the category at all was considered a nonmatch. In data-model cases, discussion was needed for alignment, but no complex situations developed. During the first screening, after discussion by the research team, 71 articles were excluded from the review for the following four reasons: (1) EHR was not a key factor but a contextual factor in the original research setting; (2) the original research did not focus on semantic interoperability but on another level of interoperability; (3) the original study did not entail practical implementation goals, but the focus was predominantly theoretical or methodological; and (4) the original research was not a research article but, for example, a poster. The remaining 58 articles were sought for retrieval. For 4 articles, the full text was not available. To evaluate eligibility, full texts of the 54 remaining articles were read by the research team. At this point, 17 articles were excluded because the original research was out of scope, that is, semantic interoperability was not developed with practical goals for advancing the availability and use of interoperable patient data. In addition, 15 articles were excluded as the semantic interoperability case did not involve EHR use or development, 3 articles were excluded due to the absence of semantic interoperability altogether, and 5 more were excluded because they were not research articles. After agreeing upon the final exclusion within our research team, 14 articles were analyzed for semantic interoperability in EHRs. Our final inclusion criteria were grounded on our research questions: the research article should explore an EHR use or development case with the focus on semantic interoperability of clinical data. Preferably, the case would document the stage of interoperability development or use, expected or realized clinical benefits, semantic development goals, and aspects of interoperability to be implemented, as well as the method of application.

**Figure 2.**
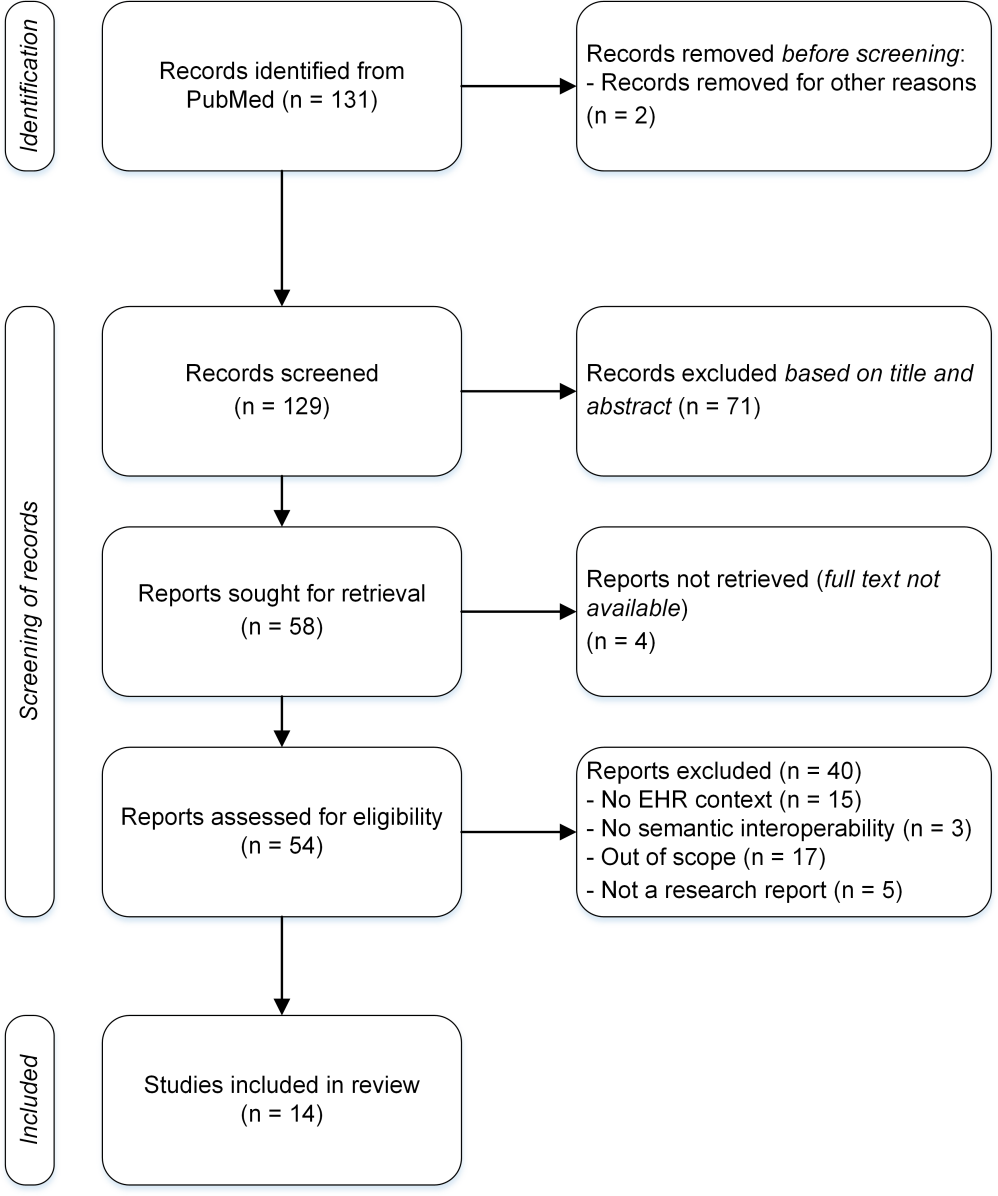
Flowchart of article identification, screening, and final inclusion. EHR: electronic health record.

The extraction and documentation of the information from the research articles was informed by our research questions, the review framework (Figure 1), and by previous research literature. At this stage, previous reviews [16-19,21] were especially used in compiling our study framework (see Figure 1). Based on our framework, the documentation of the review analysis included elements of interoperability already identified in the search strategy. Consequently, it was necessary to investigate which documented elements are typically examined in research and with what methods they are applied in EHRs [8,16-18]. Moreover, we deemed it important to document how semantic interoperability is described in the clinical use context, consisting of various EHRs, clinical applications, registers, and other data resources. Lastly, the information documentation had to include not only the semantic implementation, use goals, or intended benefits but also practical goals or benefits in the clinical use context (see Figure 2). We defined and agreed upon the information documentation categories within our research team to conduct a well-grounded analysis for the review.

## Results

### Contextual Results

We identified 14 articles describing semantic interoperability in EHRs, published between 2011 and 2022, as shown in Multimedia Appendix 1 [24-37]. The results revealed predominantly European advances in the study topic. Most (n=11, 79%) of the research cases were affiliated with different types of institutions in the European Union member states or in European Economic Area countries. One of the publications was coproduced by authors from Columbia and Germany, and the authors of another article represented organizations from the United States, South Korea, China, and Egypt. We decided not to limit the included studies to a certain geographical area but to analyze any potential use case for enabling the interoperability of EHRs.

Two of the reported research cases focused on patients with heart failure [24,30], 1 focused on patients with neurosurgical tumors [28], 2 focused on patients in cancer care [33,37], and 1 focused on patients with type 1 diabetes [31]. Other clinical use domains described were a prehospital unit at the site of an incident or during transfer to the emergency department and a hospital emergency care unit where prehospital patient documentation must be reassessed. A primary care–related case documented experimental laboratory test results of a population of 230,000 patients. Examples of older adult medication care and multiprofessional health care were part of our sample. Two articles described multipurpose clinical use of physician’s notes and tertiary care data. One article concerned the domain of clinical research using data from different EHR systems, and another described semantic aspects for retrieval of medication, laboratory test, and diagnosis-related data.

Although all studies concerned data from the EHRs, some studies included more detailed descriptions on data sources. Heart failure summaries containing clinical situation data and diagnoses (severity and certainty), as well as heart failure summaries covering clinical situations and symptoms data (a symptom’s presence, absence, and severity), were represented in the sample. One study regarded clinical history, observations, and findings during tumor control. One study focused on histories of patients with diabetes and diabetes care plans (eg, insulin regimen, diet, and exercise plans) and patients’ self-monitoring of vital signs, and 1 study used self-monitoring data on daily activities, side effects, and patient-reported outcomes. One article reported results around diagnosis and laboratory data; 1 article reported on medication, laboratory, and diagnosis data; and another article reported on neurosurgical imaging and laboratory data, although the starting point in the paper was diagnosis and medication data. The remaining 4 studies generally applied prehospital patient case data, emergency care–related EHR data, laboratory data, and diagnosis data.

### Interoperability Results

In our sample, data were transferred and shared between different EHRs and clinical applications with no loss of data or changes in their meaning (Multimedia Appendix 2 [24-37]). Half (7/14, 50%) of the studies were aimed at developing semantic interoperability between different EHRs or within different EHR modules, such as a medication module in 1 EHR system. One case concentrated specifically on an EHR and a clinical application. Two articles reported results about the interoperability between EHRs and personal health records. Interoperability with the laboratory system and the EHR was the focus of study in 2 cases. Two studies reported advances in interoperability development between EHR and clinical research resources or a clinical registry. Regarding the state of development, the largest number of studies were categorized as “in development” (n=5, 36%) and “in use” (n=6, 43%). Two articles reported results regarding the testing phase, and the remaining study was in an implementation stage.

All articles reported clinical benefits of the selected approach to enhancing semantic interoperability. We identified 3 main categories of clinical benefits within the articles: increasing the availability of data for clinicians (n=6, 43%), increasing the quality of care (n=4, 29%), and enhancing clinical data use and reuse for varied purposes (n=4, 29%). The first category describes use cases where patient care would benefit from better availability of data. This was to be achieved by enhancing interoperable data and its transfer from clinical applications (eg, a laboratory system) to a central EHR and between EHRs to increase accessible data for making informed clinical decisions. These advances were in implementation to enhance the quality and effectiveness of care. Moreover, developing better access to health data and providing homogeneous access to heterogeneous data sets may facilitate resource effectiveness; patient management; and overall, the optimization of data for different purposes. The second category included benefits for the quality of care. The category had largely been implemented in EHRs already. Benefits entail better resource effectiveness and optimization of patient care planning and monitoring and better patient management, as well as the continuity of care based on interoperable and accessible health data that facilitates informed decision-making by clinicians. One of these cases documented improved patient safety based on interoperable health data across EHRs. The third category, enhancing clinical data use and reuse, included 2 use cases where data were used across EHRs. One use case described data transfer between an EHR and a national oncology registry, where interoperability enhanced data integration and redesign of the systems in use. The other 2 cases documented the evidence of data use, where better availability of data provided a means for developing new EHR integrated tools, such as clinical alerts, dynamic patient lists, and clinical follow-up dashboards. In summary, semantic development goals emphasized better access to data regardless of underlying standards and data structures or EHRs in use. The underlying assumption is that with better access to data, it is possible to facilitate better communication between professionals and the continuity of care.

In our analysis, semantic development goals were divided in 3 categories. All of these were closely coupled with the need to build usable and available data based on heterogeneous medical information that is accessible through various EHRs and databases, such as registers. Data harmonization and developing semantic interoperability between different EHRs or between EHRs and clinical application was the largest category (n=8, 57%). Enhancing health data quality through standardization (n=5, 36%) and developing EHR-integrated tools based on interoperable data (n=1, 7%) were the other identified categories. Semantic development goals were described as harmonizing data or otherwise processing semantically equivalent data across different medical domains and among different clinical data sources including EHRs and applications, thus facilitating clinicians’ availability of health data. One case included the formalization of data with a semantic converter to increase the interoperability of data. In 2 research cases, the main semantic development goals concentrated on advancing the interoperability of EHR data and patient-generated data or sensor data to monitor the situation of patients who are chronically ill. Regarding data standardization, 1 research case reported increasing data quality as the semantic interoperability development goal. Standardized data content decreased information overload of clinicians. Through data standardization, it was possible to increase conceptualization and, thus, access to data within an EHR regardless of the underlying standards and data structures, by providing a semantic standardized layer to facilitate clinicians’ data use, or by otherwise ensuring complete and coherent information with no errors due to the loss of meaning or context. One of these research cases documented improvements for system-level efficiency for EHR functions and integrated tools based on advances of semantic interoperability.

Features of semantic interoperability were described in all 14 articles. Most (9/14, 64%) of the analyzed cases incorporated 1 or more semantic aspects. In more detail, the aspects of semantic interoperability were described as follows: ontologies were the chosen aspect in 3 research cases, terminologies in 6 cases, classifications in 4 cases, various clinical documentation standards in 8 cases, and different data models in 10 cases. In this categorization, data model refers to various semantic model layers, namely, the use of various types of data models that include, for example, data content specifications, RIMs, and clinical information models depending on the development context. A dual model was discussed in 2 of the cases for the application of data models.

Closely related to the aspects of interoperability, several interoperability standard solutions were named. Named ontology solutions included a top-domain ontology for the life sciences (BioTopLite) in 2 cases, a HL7 Fast Health Interoperability Resources (FHIR) and semantic sensor network–based type 1 diabetes ontology for type 1 diabetes data, and a system of several ontologies to be used for building EHR interoperability. Systematized Nomenclature of Medicine Clinical Terminology was the common terminology application in 7 cases, whereas classification systems were applied in more heterogeneous ways. The following international classifications were named: *International Classification of Diseases, Tenth Revision*; *International Classification of Diseases, Ninth Revision, Clinical Modification*; The Anatomical Therapeutic Chemical Classification System; and Logical Observation Identifiers Names and Codes. One article documented national classification use. Applied health care–specific standards included the open standard specification in health informatics (openEHR; n=6), Digital Imaging and Communications in Medicine (n=1), HL7 FHIR (n=5), and the HL7 Clinical Document Architecture (n=2). Regarding data models or reference information models, several types were applied for distinct use environments. These included the Observational Medical Outcomes Partnership common data model, an EHR-specific data component model, the i2b2 common data model for data warehouse development, the HL7 FHIR RIM, and the EN/ISO 13606 standard–based model. Moreover, 1 case reported using openEHR as a data model reference.

The method for applying an interoperability framework or approach is related to the overall design of the data use purposes and the needs driving the semantic development. The chosen methodology for semantic development was based on ontology development or the application of an ontology framework in 4 research cases, data model–based development in 5 cases, archetype development in 1 case, and clinical data warehouse development to enhance access and processing of data in 1 case. In data model–based approaches, use cases document a method’s capability in separating different semantic levels of development, that is, system level, application level, clinical user interface level, or patient information level. The reusability of data model–based semantic approaches and related methods were assessed for resource savings in time and cost in development projects and, thus, to justify the choice of the approach. For example, clinical knowledge model–based development may allow recycling archetypes that further promote semantic interoperability.

## Discussion

### Principal Findings

Our results are related to the main goals of semantic interoperability development, such as enabling patient data use regardless of which EHR the data originated from and by which terminologies, classifications, or other semantic features they are supported [16-19,21]. Regarding key elements of semantic interoperability, of the documented terminologies, Systematized Nomenclature of Medicine Clinical Terminology seemed to prevail as the dominant choice for clinical terminology [24-30]. For international classifications that are typically integrated into EHRs, a selection of well-established classifications was documented [25,26,31,32]. Likewise, several health care specific standards [24-26,28,31,33], ontologies [21,24,32,33], and data models [25,27,28,30-36] were presented, albeit in a relatively small sample in this study. One possible factor affecting the selection of interoperability features such as international standards may be open availability and the level of cost of the standard-specific resources and their deployment. Consequently, shared implementation experiences and recommendations from previous projects or from collaboration in international communities may promote and facilitate decision-making concerning future implementations.

Our review illustrates several approaches for building sematic interoperability. For ontologies and data models, based on the review, several layers may be deployed to address semantic interoperability development needs. For ontologies, deploying a system of ontologies seeks to bridge, for example, domain-specific ontologies and application-specific ontologies. In our sample, a case with a data model–based development approach enhanced the communication of clinical information with the application. The application was used by the patients in self-monitoring, and the EHR served as a clinical data repository to avoid the loss of meaningful information. In general, when applying data model–based approaches, a dual model or multimodel approach may be needed to address different semantic interoperability issues during development—from the clinician as an EHR user to the system transaction level.

Our review highlights several clinical benefits of semantic interoperability. Primarily semantic interoperability fulfills the need to support the implementation of applications that enhance the continuity of care and ensure access to safe and high-quality health care. The reported clinical benefits of developing semantic interoperability reflect well common international goals [2,3,5]. The results in our sample show that an evident goal driving the development in these studies is the following assumption: through increased access to patient information, better quality and outcomes in care can be achieved [24,26,27,33,37]. Better communication based on easily accessible data across EHRs is facilitated not only between clinicians but also between professionals and patients [28,34,35]. Further advances are related to efficiency and subsequent economic factors, for example, reducing the clinicians’ workload for documenting and evaluating extensive patient data, to avoid information overload and support multiprofessional care [26,31-33,35]. In addition, interoperable patient data provide opportunities for a wide range of EHR-related clinical development, for example, regarding decision-making support, other EHR integrated tools, clinical research, or other types of secondary use [25,28-31,33,36]. Essentially, the interoperability cases in our review demonstrated a well-documented selection of development goals in EHRs, including considerations of patient-generated, self-monitoring data and related interoperability features.

Finally, when reflecting on the goal-related semantic interoperability results, there is evidently not one universal approach available to tackle all interoperability-related needs and challenges. One reason for this is that interoperability is to be achieved in diffuse and disconnected clinical care settings and in registry data use across borders. However, regulations and international recommendations can support the choosing of common tools and standards for building interoperability for patient data generated in various EHRs and clinical applications. This may be the strongest selling point for evolving international frameworks, such as the EHDS regulation proposal. If adopted, unified toolkits of the most crucial means can be achieved for building international eHealth interoperability. Through these mechanisms, common solutions and standards can be agreed upon to remedy existing inconsistencies and avoid possible future imparities that hinder the realization of the common goals. It is noteworthy that all member states have steps to take to meet the international requirements with a country-specific road map to achieve the common goal [3,5]. Moreover, it would require cooperation to align on which level of interoperability should be reached when the operating environment consists of a diverse set of clinical practices and related data needs, such as between public and private care or between primary and specialized care. Additionally, it may be worthwhile to consider whether instead of promoting a single approach, semantic interoperability requirements should be assessed through several levels of semantic requirements, such as standards, data models, classifications, and terminologies. Moreover, developing the necessary skills and increasing capabilities is an essential component of this development.

Specifically, regarding European development, one of the main goals is to support the use of health data for better health care delivery and better research. The comprehensive and timely availability of EHR data is known to improve the quality of care and patient safety [26,38]. Concurrently, the lack of not only technical or organizational but also semantic interoperability has been recognized as one of the barriers for the cross-border exchange of health data [2-8]. Therefore, commonly recognized interoperability approaches and standards for the harmonization of semantic interoperability are needed.

### Limitations

Our goal was to ensure that we did not overlook any important studies and to minimize any potential biases by conducting a thorough and comprehensive search of the available literature. However, it is worth noting that our search was limited to a single database, PubMed. Nevertheless, recent literature suggests that PubMed can serve as a primary search tool. It possesses the necessary capabilities for systematic reviews, including the ability to formulate and interpret queries accurately, as well as ensuring search reproducibility. It is important to acknowledge that even a well-performing system such as PubMed might not always yield the desired results in different scenarios [23]. Our data set was limited by a small sample size of 14 articles. Therefore, findings can only be regarded as descriptive in nature. Relatively large heterogeneity in study environments and selected research approaches limit us from drawing strong conclusions. Despite these limitations, this review demonstrates potentially feasible approaches for promoting semantic interoperability toward harmonized approaches. Additional real-world studies accounting for semantic interoperability are needed to reinforce understanding of the most promising, scalable examples such as international reference models (eg, HL7 RIM). Moreover, it was challenging to determine the “development status” category for certain studies. This was due to varying levels of details in the study reports, where some of the studies provided a wealth of detail, whereas some were more restricted in their scope.

### Suggestions for Future Research

Future research directions are 2-fold from the current development perspective. First, evidence-based recommendations on semantic interoperability features, for example, data models and terminologies, are needed. Initially, the applicability of international data models and standards such as HL7 V2 might be evaluated. Second, more experiences of interoperability development should be reported in the peer-reviewed research literature to contribute evidence around successful and not so successful experiences instead of leaning solely on individual expert opinions. Presumably, due to the evolving implementation status of semantic interoperability cases illustrated in the research literature, systematic research–based evaluation of benefits and outcomes is still scarce.

### Conclusions

We conclude that based on our review, the research literature highlights valuable aspects in promoting semantic interoperability in terms of the efficiency and feasibility of solutions integrated in EHRs and possibly for enhancing care. However, when heading toward semantic harmonization, more data, pilot experiences, and analyses are needed to assess how applicable the chosen specific solutions are for the standardization and semantic interoperability of patient data. Instead of promoting a single approach, semantic interoperability could be assessed through several levels of semantic approaches. A dual model or multimodel approach is usable to address different semantic interoperability issues during development—from the clinician as an EHR user to the system transaction level. Since interoperability is being implemented in complex and disconnected clinical care environments, choices should be well prepared and justified to meet sustainable and long-term outcomes. From that point of view, it is possible to outline future directions in selecting semantic interoperability approaches for the realization of the international patient data–related goals.

## Supplementary material

10.2196/53535 Multimedia Appendix 1 Summary of study and sample characteristics.

10.2196/53535 Multimedia Appendix 2 Summary of results on semantic interoperability in electronic health records.

10.2196/53535 Checklist 1 PRISMA (Preferred Reporting Items for Systematic Reviews and Meta-Analyses) checklist.

## Abbreviations

EHDS: European Health Data Space
EHR: electronic health record
EIF: European Interoperability Framework
FHIR: Fast Health Interoperability Resources
HL7: Health Level 7
RIM: reference information model
WHO: World Health Organization
