# Supplementary material for: Semantic Interoperability of Electronic Health Records: Systematic Review of Alternative Approaches for Enhancing Patient Information Availability
Source: JMIR Med Inform. 2024 Apr 25;12:e53535. doi: 10.2196/53535 (PMC11066539; doi:10.2196/53535)
Supplement: Multimedia Appendix 1 [file medinform-v12-e53535-s001.docx]

Multimedia Appendix 1. Summary of study and sample characteristics [24-37].

| Reference | Journal | DOI | Year of publication | Country | Clinical use domain | Data sources |
| --- | --- | --- | --- | --- | --- | --- |
| 24 | Appl Clin Inform | 10.4338/ACI-2014-04-RA-0031 | 2014 | Austria, Germany | heart failure patients; tobacco use | heart failure summary; clinical situation data and diagnosis (severity, certainty) |
| 25 | J Biomed Inform | 10.1016/j.jbi.2015.10.009 | 2015 | Belgium | clinical research | health care data; diagnosis and laboratory results |
| 26 | Stud Health Technol Inform | 10.3233/SHTI210149 | 2021 | Denmark | prehospital and hospital emergency care | prehospital (patient case data) and emergency care data (EHR data) |
| 32 | Stud Health Technol Inform | 10.3233/978-1-60750-806-9-694 | 2011 | Germany, Columbia | multidisciplinary health care | laboratory data |
| 33 | Appl Clin Inform | 10.4338/ACI-2017-01-RA-0009 | 2017 | Germany | neurosurgical tumor patients | neurosurgery patient records; imaging and laboratory data |
| 34 | Int J Med Inform | 10.1016/j.ijmedinf.2015.05.016 | 2015 | Norway, Spain | primary care | laboratory data |
| 27 | J Am Med Inform Assoc | 10.1093/jamia/ocu013 | 2015 | Austria, Netherlands, Sweden, UK | heart failure patients | heart failure summary; clinical situation and symptoms data (symptom’s presence, absence, and severity) |
| 35 | BMC Med Inform Decis Mak | 10.1186/s12911-019-0806-z | 2019 | Egypt, South Korea, USA | type 1 diabetes patients; self-monitoring | patient history and diabetes care plan (e.g., insulin regimen, diet, and exercise plan); monitoring data (vital signs) |
| 31 | BMC Med Inform Decis Mak | 10.1186/s12911-017-0513-6 | 2017 | France | elderly care | patient record data; diagnosis and medication |
| 28 | JMIR Med Inform. | 10.2196/40344 | 2022 | Spain | cancer care patients; self-monitoring | monitoring data; daily activity, side effects, and PROMs |
| 37 | J Am Med Inform Assoc. | 10.1136/amiajnl-2012-000855 | 2012 | Sweden | patient data in multiprofessional use context | physicians’ notes |
| 29 | Stud Health Technol Inform | 10.3233/978-1-61499-678-1-436 | 2016 | Denmark | oncology data, head and neck cancer | patient data, e.g., clinical history, observations and findings during tumor control, side effects to therapy |
| 30 | Stud Health Technol Inform. | 10.3233/SHTI210114 | 2021 | Spain | tertiary hospital data | observations for SARS-COV-2 diagnostic tests and clinical alerts necessary to identify infected and at-risk patients |
| 36 | J Med Internet Res | 10.2196/13504 | 2019 | China | patient data retrieval | medication, laboratory test, and diagnosis data |
