# Supplementary material for: Semantic Interoperability of Electronic Health Records: Systematic Review of Alternative Approaches for Enhancing Patient Information Availability
Source: JMIR Med Inform. 2024 Apr 25;12:e53535. doi: 10.2196/53535 (PMC11066539; doi:10.2196/53535)
Supplement: Multimedia Appendix 2 [file medinform-v12-e53535-s002.docx]

Multimedia Appendix 2. Summary of results on semantic interoperability in EHRs [24-37].

| Reference | EHR interoperability | State of development | Clinical benefits | Semantic goals | Aspects of interoperability | Named standards of interoperability | Method of application |
| --- | --- | --- | --- | --- | --- | --- | --- |
| 24 | EHR–EHR | in development | increasing availability of data | data harmonization | ontology  terminology  standards | BioTopLite ontology  SNOMED CT  openEHR, HL7 CDA (tobacco use models) | application of ontology framework |
| 25 | EHR–clinical research resources | in testing towards implementation | enhancing clinical data use and re-use | data harmonization | data model (data content)  classification  terminology  standard | OMOP Common Data Model  ICD-10  ICD-9-CM  ATC  LOINC  SNOMED CT  HL7 CDA | data model development |
| 26 | EHR–clinical application | in development | increasing availability of data | enhancing data quality by standardization | terminology  standard | SNOMED CT  HL7 FHIR | archetype development |
| 32 | EHR–integrated laboratory system | in use and prototype in testing | increasing quality of care | data harmonization | data model  ontology | generic component model  system of ontologies | ontology-based development |
| 33 | EHR–EHR | in use | increasing availability of data | data harmonization | data model  reference information model  standard | openEHR  DICOM  HL7 RIM | data model-based development |
| 34 | EHR–laboratory system–clinical research resources | in testing | increasing availability of data | enhancing data quality by standardization | clinical in-formation model  reference information model | openEHR | archetype development |
| 27 | EHR–EHR | in development | increasing availability of data | enhancing data quality by standardization | information models  terminology  ontology | BioTopLite2 ontology  openEHR SNOMED CT | application of ontology framework |
| 35 | EHR–PHR^a^ | in development | increasing quality of care | data harmonization | data model  ontology  standard | T1D Ontology (FASTO)  HL7 FHIR | ontology-based development with logical data models |
| 31 | EHR–EHR medication module | in implementation | increasing availability of data | data harmonization | data model  classification  standard | i2b2 data model ATC  ICD  HL7 FHIR | clinical data warehouse development |
| 28 | EHR–PHR^a^ | in use | increasing quality of care | data harmonization | data model classification terminology standard | ISO EN 13606, openEHR as data models  ICD-10  LOINC  SNOMED CT  HL7 FHIR | data model-based development |
| 37 | EHR-EHR | in use | increasing quality of care | developing EHR integrated tools | structuring EHR data  classification | national coding systems | structuring EHR data |
| 29 | EHR-national oncology registry | in use | enhancing clinical data use and re-use | data harmonization | terminology | SNOMED CT | conceptualization of data |
| 30 | EHR-EHR | in use | enhancing clinical data use and re-use | enhancing data quality by standardization | data model  standards | i2b2 data model  LOINC  SNOMED CT | conceptualization of data |
| 36 | EHR-EHR | in development | enhancing clinical data use and re-use | enhancing data quality by standardization | clinical information models CIMs | openEHR | data model development |

^a^Personal health record
